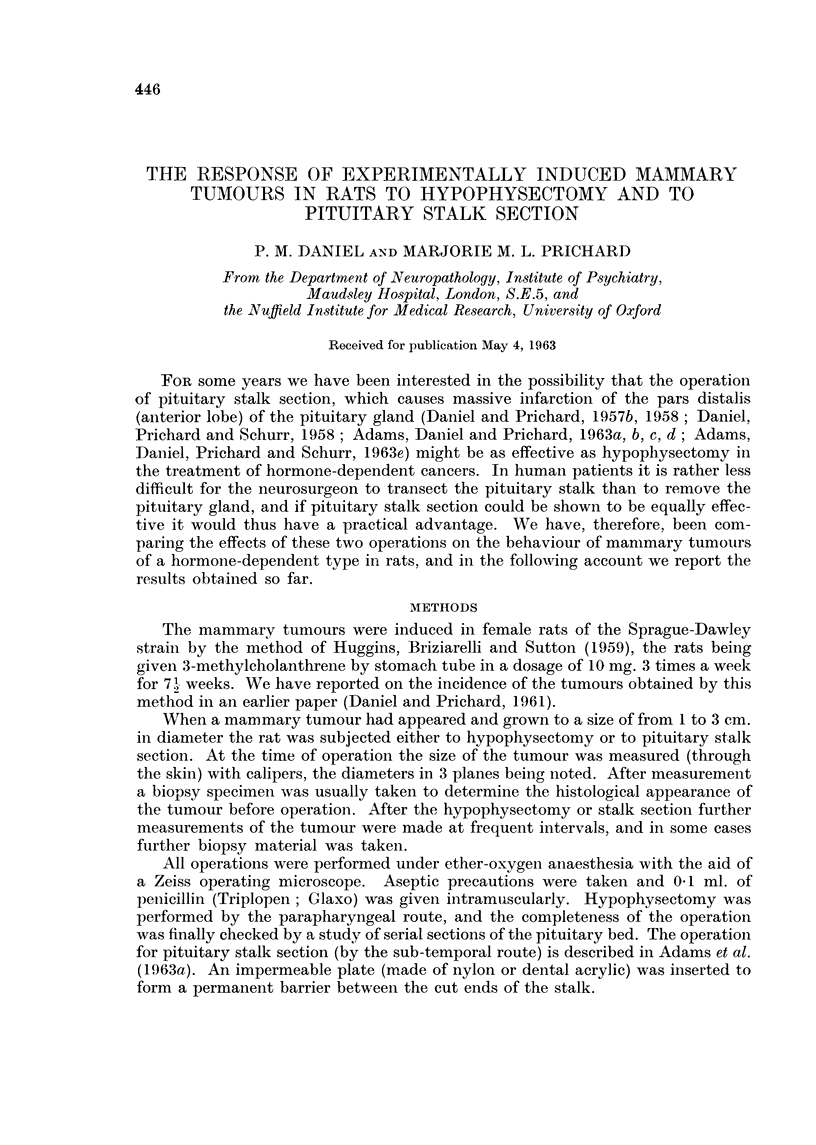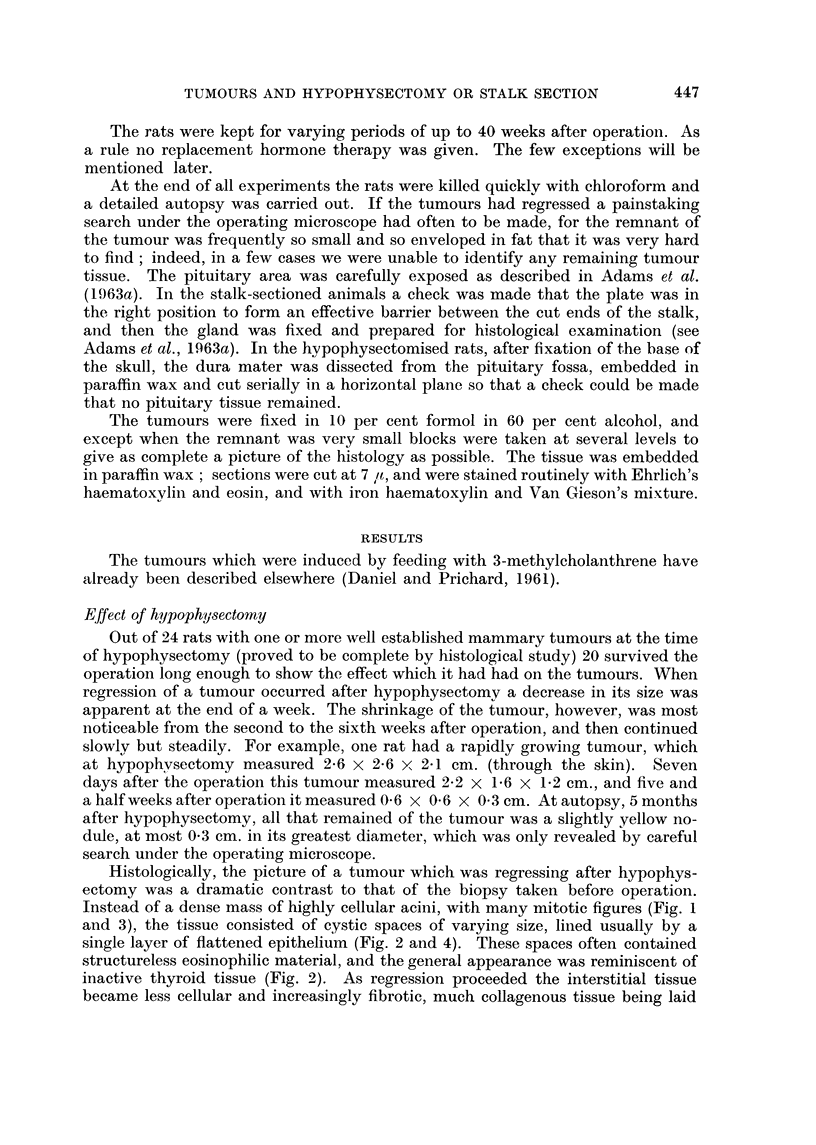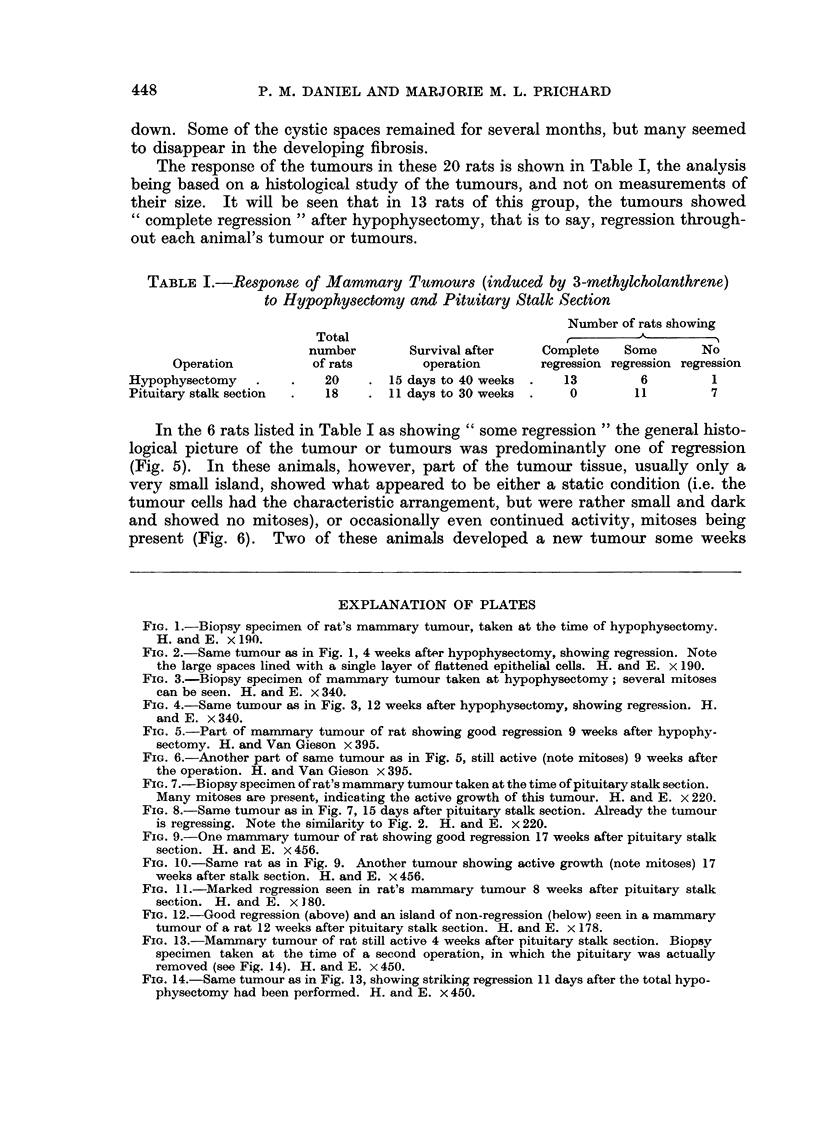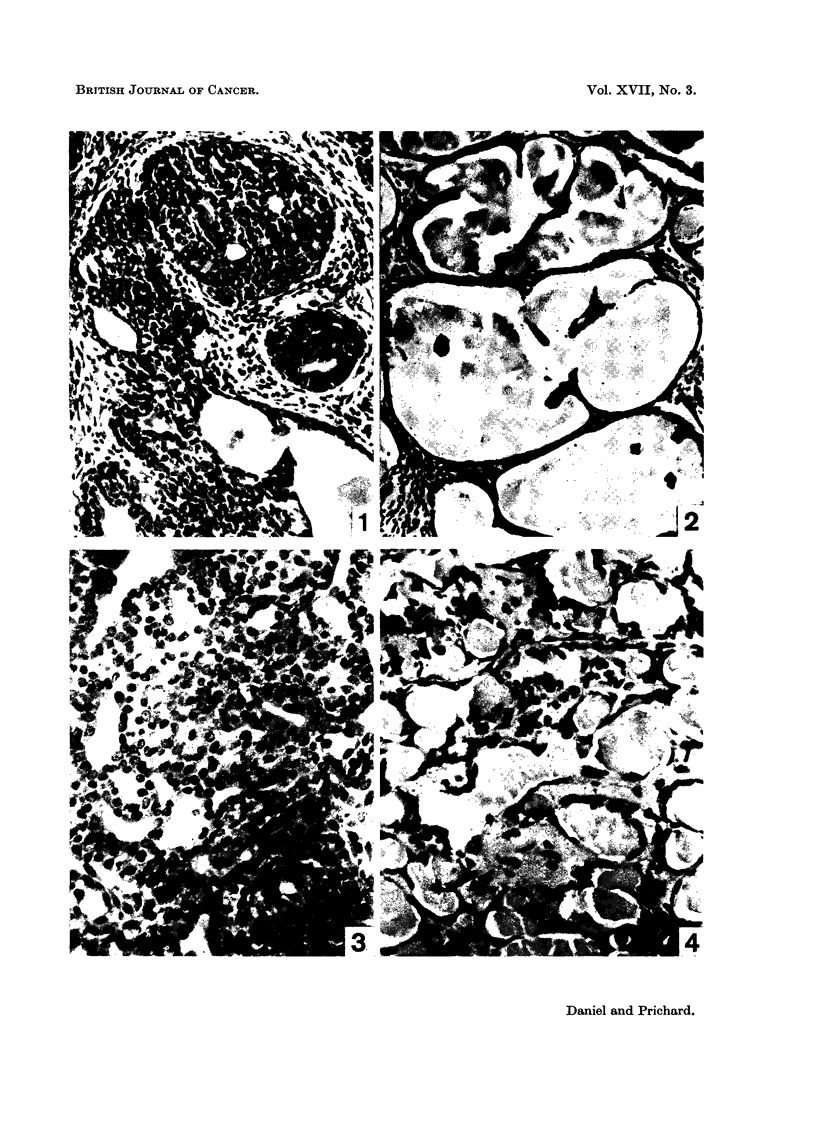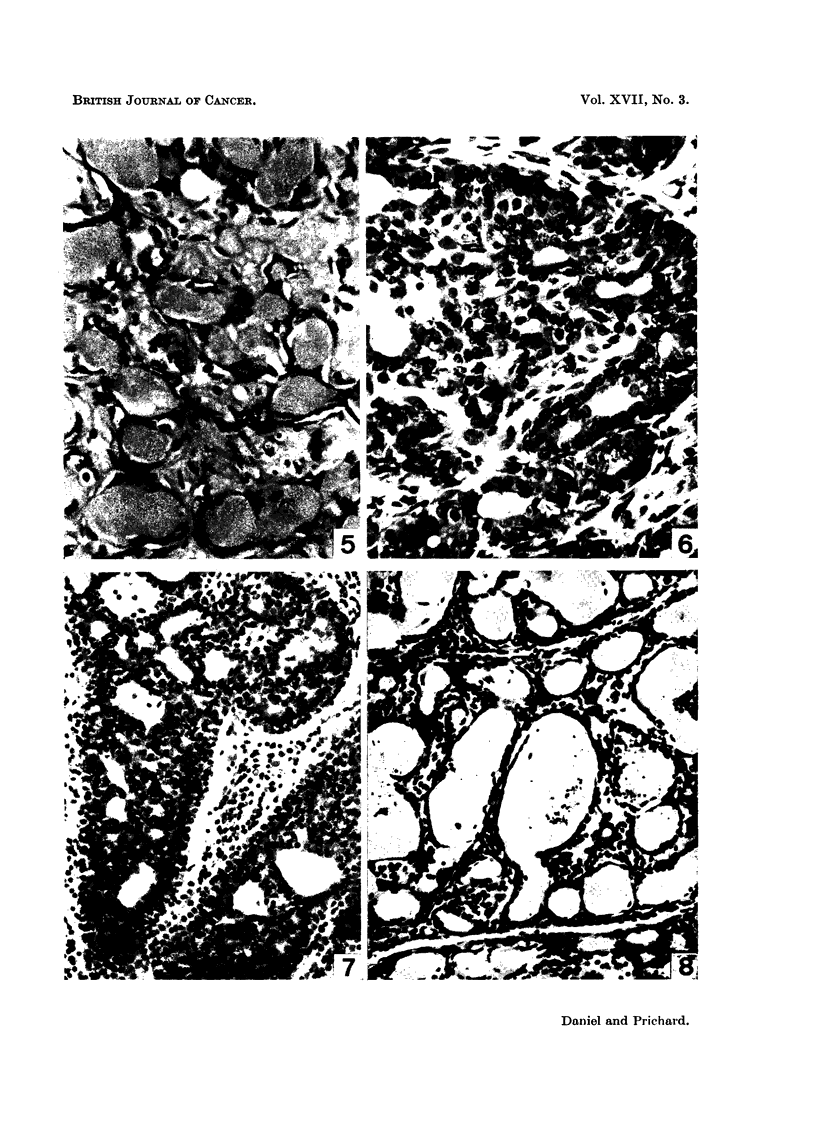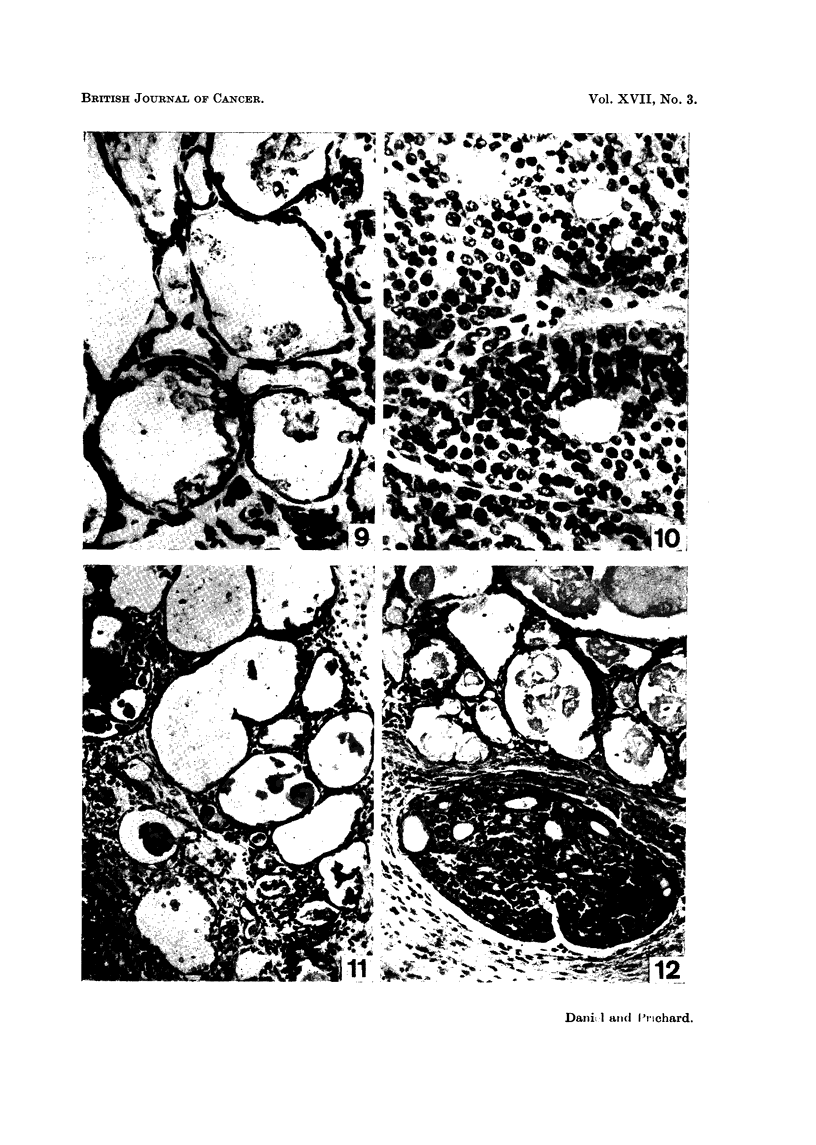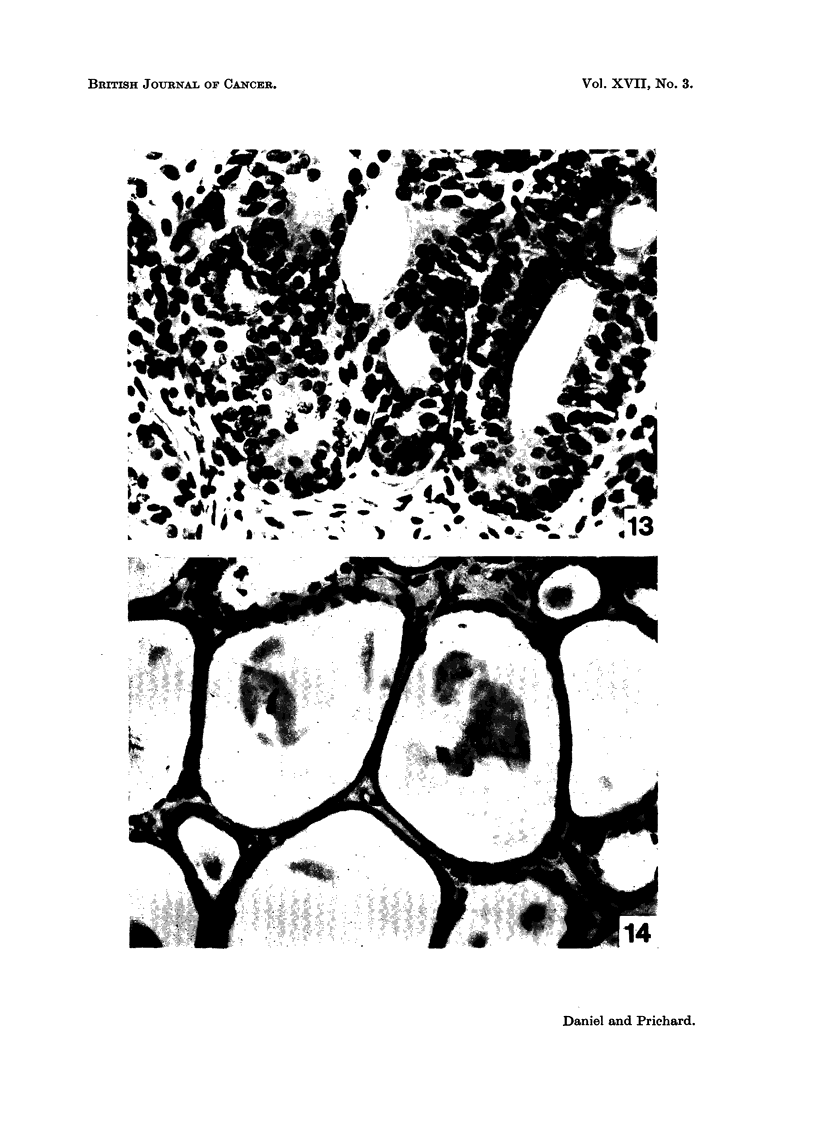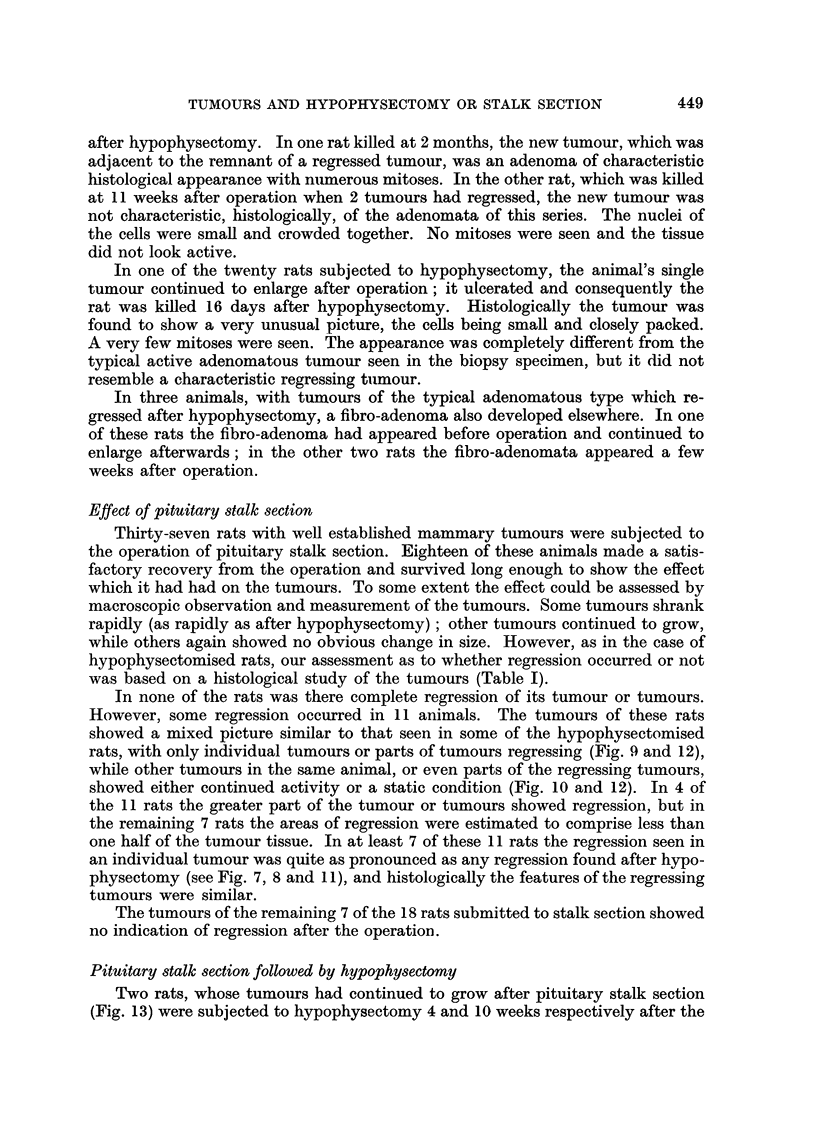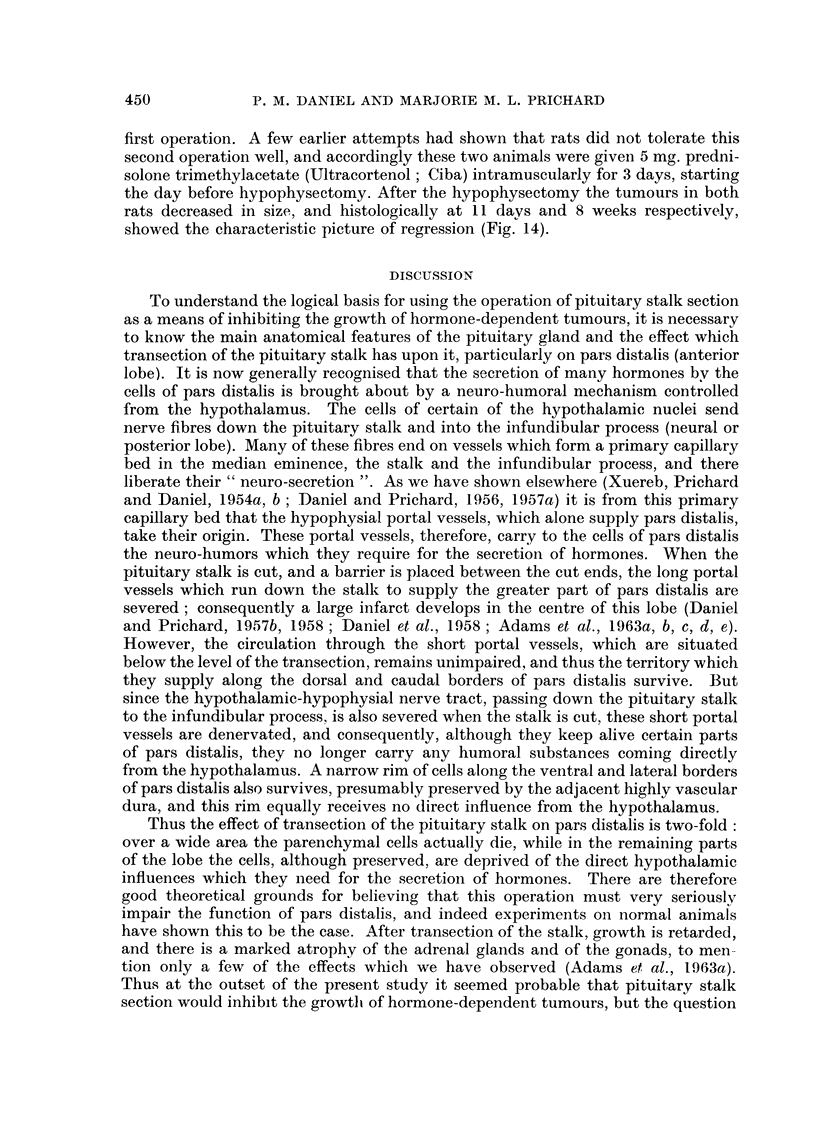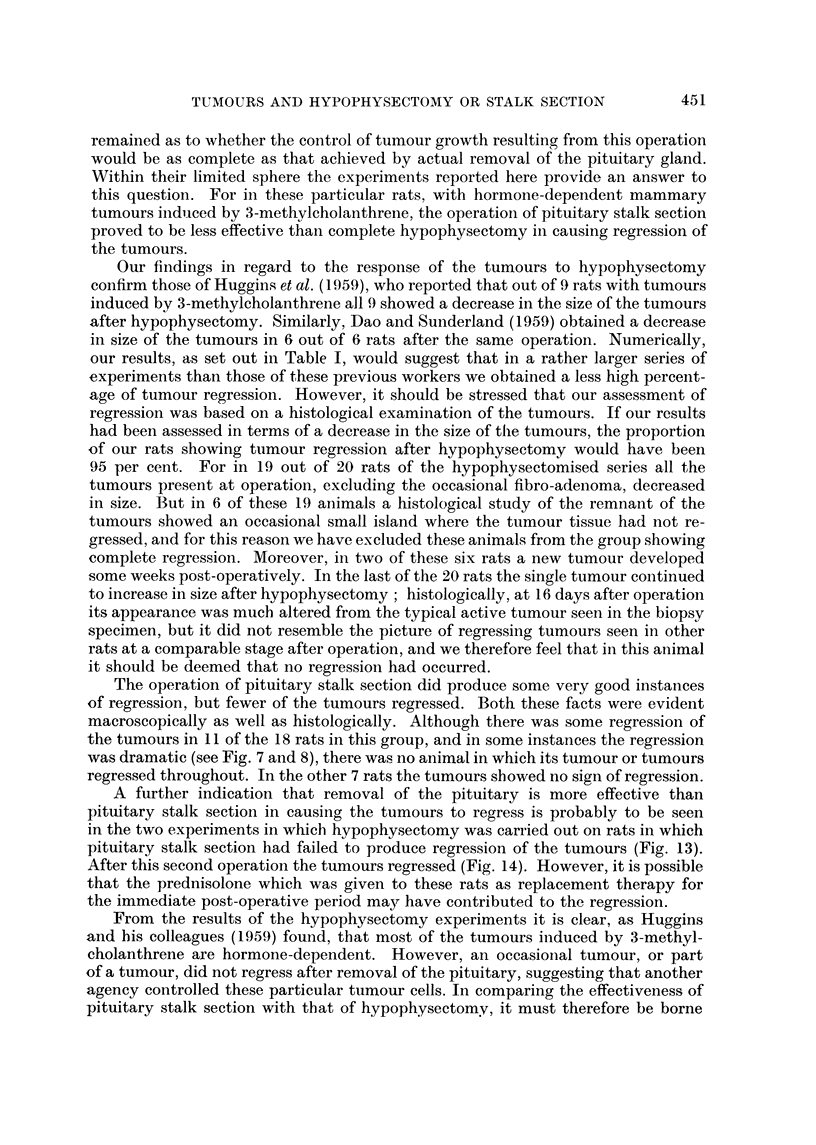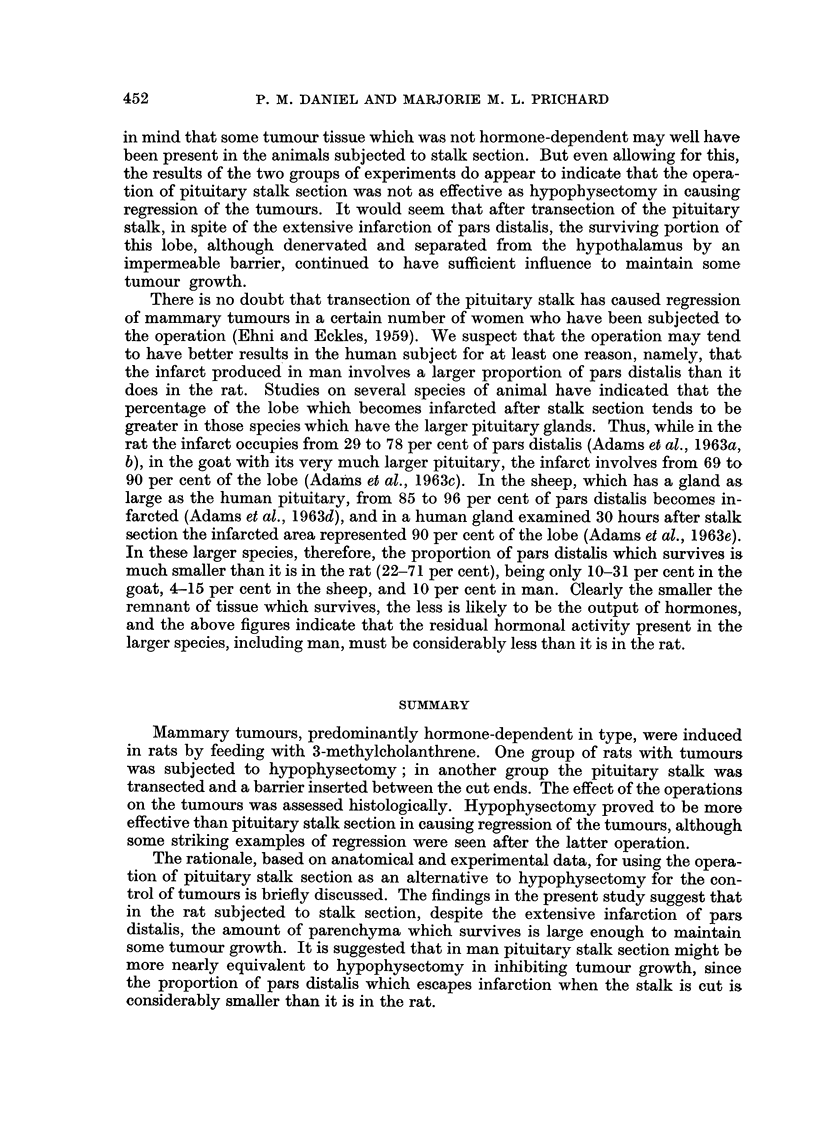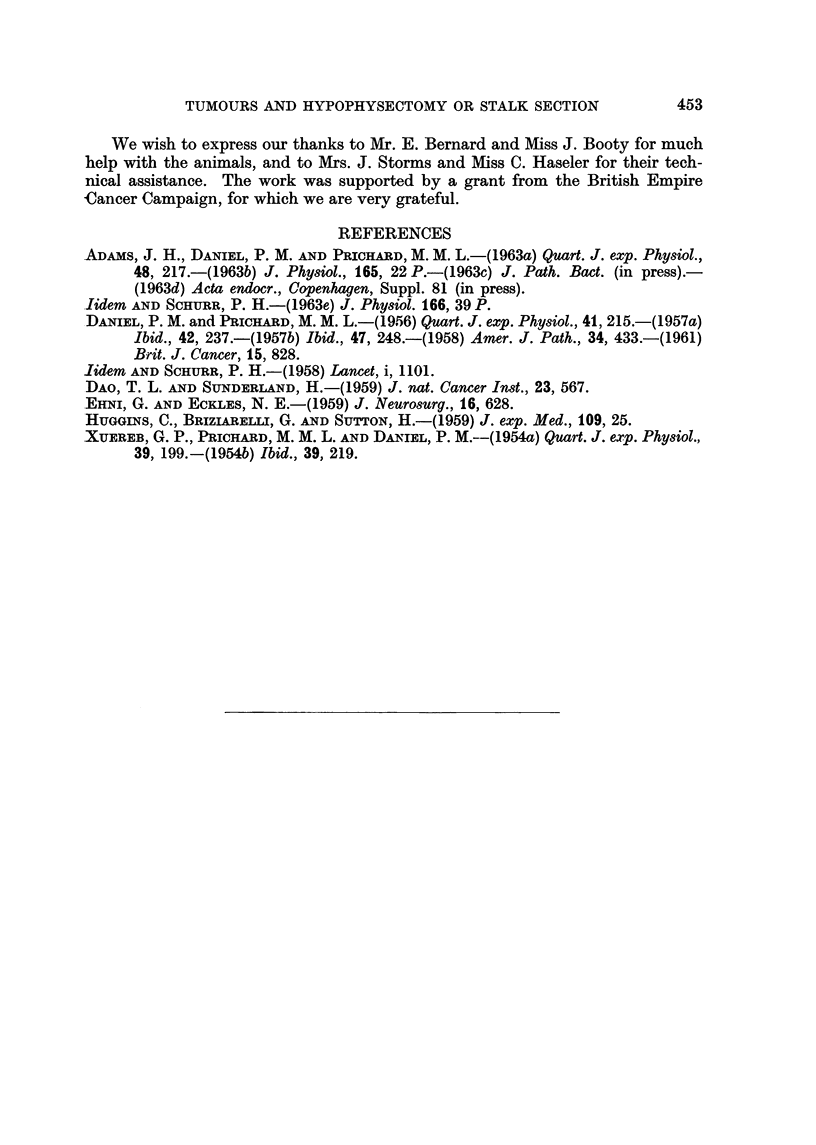# The Response of Experimentally Induced Mammary Tumours in Rats to Hypophysectomy and to Pituitary Stalk Section

**DOI:** 10.1038/bjc.1963.59

**Published:** 1963-09

**Authors:** P. M. Daniel, Marjorie M. L. Prichard

## Abstract

**Images:**


### THE RESPONSE OF EXPERIMENTALLY INDUCED MAMMARY TUMOURS IN RATS TO HYPOPHYSECTOMY AND TO PITUITARY STALK SECTION

P. M. DANIEL AND MARJORIE M. L. PRICHARD

*From the Department of Neuropathology, Institute of Psychiatry, Maudsley Hospital, London, S.E.5, and the Nuffield Institute for Medical Research, University of Oxford*

Received for publication May 4, 1963

FOR some years we have been interested in the possibility that the operation of pituitary stalk section, which causes massive infarction of the pars distalis (anterior lobe) of the pituitary gland (Daniel and Prichard, 1957*b*, 1958 ; Daniel, Prichard and Schurr, 1958 ; Adams, Daniel and Prichard, 1963*a*, *b*, *c*, *d* ; Adams, Daniel, Prichard and Schurr, 1963*e*) might be as effective as hypophysectomy in the treatment of hormone-dependent cancers. In human patients it is rather less difficult for the neurosurgeon to transect the pituitary stalk than to remove the pituitary gland, and if pituitary stalk section could be shown to be equally effective it would thus have a practical advantage. We have, therefore, been comparing the effects of these two operations on the behaviour of mammary tumours of a hormone-dependent type in rats, and in the following account we report the results obtained so far.

### METHODS

The mammary tumours were induced in female rats of the Sprague-Dawley strain by the method of Huggins, Briziarelli and Sutton (1959), the rats being given 3-methylcholanthrene by stomach tube in a dosage of 10 mg. 3 times a week for $7\frac{1}{2}$ weeks. We have reported on the incidence of the tumours obtained by this method in an earlier paper (Daniel and Prichard, 1961).

When a mammary tumour had appeared and grown to a size of from 1 to 3 cm. in diameter the rat was subjected either to hypophysectomy or to pituitary stalk section. At the time of operation the size of the tumour was measured (through the skin) with calipers, the diameters in 3 planes being noted. After measurement a biopsy specimen was usually taken to determine the histological appearance of the tumour before operation. After the hypophysectomy or stalk section further measurements of the tumour were made at frequent intervals, and in some cases further biopsy material was taken.

All operations were performed under ether-oxygen anaesthesia with the aid of a Zeiss operating microscope. Aseptic precautions were taken and 0·1 ml. of penicillin (Triplopen ; Glaxo) was given intramuscularly. Hypophysectomy was performed by the parapharyngeal route, and the completeness of the operation was finally checked by a study of serial sections of the pituitary bed. The operation for pituitary stalk section (by the sub-temporal route) is described in Adams *et al.* (1963*a*). An impermeable plate (made of nylon or dental acrylic) was inserted to form a permanent barrier between the cut ends of the stalk.

The rats were kept for varying periods of up to 40 weeks after operation. As a rule no replacement hormone therapy was given. The few exceptions will be mentioned later.

At the end of all experiments the rats were killed quickly with chloroform and a detailed autopsy was carried out. If the tumours had regressed a painstaking search under the operating microscope had often to be made, for the remnant of the tumour was frequently so small and so enveloped in fat that it was very hard to find ; indeed, in a few cases we were unable to identify any remaining tumour tissue. The pituitary area was carefully exposed as described in Adams *et al.* (1963*a*). In the stalk-sectioned animals a check was made that the plate was in the right position to form an effective barrier between the cut ends of the stalk, and then the gland was fixed and prepared for histological examination (see Adams *et al.*, 1963*a*). In the hypophysectomised rats, after fixation of the base of the skull, the dura mater was dissected from the pituitary fossa, embedded in paraffin wax and cut serially in a horizontal plane so that a check could be made that no pituitary tissue remained.

The tumours were fixed in 10 per cent formol in 60 per cent alcohol, and except when the remnant was very small blocks were taken at several levels to give as complete a picture of the histology as possible. The tissue was embedded in paraffin wax ; sections were cut at 7 $\mu$, and were stained routinely with Ehrlich's haematoxylin and eosin, and with iron haematoxylin and Van Gieson's mixture.

### RESULTS

The tumours which were induced by feeding with 3-methylcholanthrene have already been described elsewhere (Daniel and Prichard, 1961).

### *Effect of hypophysectomy*

Out of 24 rats with one or more well established mammary tumours at the time of hypophysectomy (proved to be complete by histological study) 20 survived the operation long enough to show the effect which it had had on the tumours. When regression of a tumour occurred after hypophysectomy a decrease in its size was apparent at the end of a week. The shrinkage of the tumour, however, was most noticeable from the second to the sixth weeks after operation, and then continued slowly but steadily. For example, one rat had a rapidly growing tumour, which at hypophysectomy measured 2·6 × 2·6 × 2·1 cm. (through the skin). Seven days after the operation this tumour measured 2·2 × 1·6 × 1·2 cm., and five and a half weeks after operation it measured 0·6 × 0·6 × 0·3 cm. At autopsy, 5 months after hypophysectomy, all that remained of the tumour was a slightly yellow nodule, at most 0·3 cm. in its greatest diameter, which was only revealed by careful search under the operating microscope.

Histologically, the picture of a tumour which was regressing after hypophysectomy was a dramatic contrast to that of the biopsy taken before operation. Instead of a dense mass of highly cellular acini, with many mitotic figures (Fig. 1 and 3), the tissue consisted of cystic spaces of varying size, lined usually by a single layer of flattened epithelium (Fig. 2 and 4). These spaces often contained structureless eosinophilic material, and the general appearance was reminiscent of inactive thyroid tissue (Fig. 2). As regression proceeded the interstitial tissue became less cellular and increasingly fibrotic, much collagenous tissue being laid down. Some of the cystic spaces remained for several months, but many seemed to disappear in the developing fibrosis.

The response of the tumours in these 20 rats is shown in Table I, the analysis being based on a histological study of the tumours, and not on measurements of their size. It will be seen that in 13 rats of this group, the tumours showed " complete regression " after hypophysectomy, that is to say, regression throughout each animal's tumour or tumours.

TABLE I.—*Response of Mammary Tumours (induced by 3-methylcholanthrene) to Hypophysectomy and Pituitary Stalk Section*

| Operation | Total number of rats | Survival after operation | Number of rats showing: Complete regression | Some regression | No regression |
|---|---|---|---|---|---|
| Hypophysectomy | 20 | 15 days to 40 weeks | 13 | 6 | 1 |
| Pituitary stalk section | 18 | 11 days to 30 weeks | 0 | 11 | 7 |

In the 6 rats listed in Table I as showing " some regression " the general histological picture of the tumour or tumours was predominantly one of regression (Fig. 5). In these animals, however, part of the tumour tissue, usually only a very small island, showed what appeared to be either a static condition (i.e. the tumour cells had the characteristic arrangement, but were rather small and dark and showed no mitoses), or occasionally even continued activity, mitoses being present (Fig. 6). Two of these animals developed a new tumour some weeks

---

EXPLANATION OF PLATES

FIG. 1.—Biopsy specimen of rat's mammary tumour, taken at the time of hypophysectomy. H. and E. ×190.

FIG. 2.—Same tumour as in Fig. 1, 4 weeks after hypophysectomy, showing regression. Note the large spaces lined with a single layer of flattened epithelial cells. H. and E. ×190.

FIG. 3.—Biopsy specimen of mammary tumour taken at hypophysectomy; several mitoses can be seen. H. and E. ×340.

FIG. 4.—Same tumour as in Fig. 3, 12 weeks after hypophysectomy, showing regression. H. and E. ×340.

FIG. 5.—Part of mammary tumour of rat showing good regression 9 weeks after hypophysectomy. H. and Van Gieson ×395.

FIG. 6.—Another part of same tumour as in Fig. 5, still active (note mitoses) 9 weeks after the operation. H. and Van Gieson ×395.

FIG. 7.—Biopsy specimen of rat's mammary tumour taken at the time of pituitary stalk section. Many mitoses are present, indicating the active growth of this tumour. H. and E. ×220.

FIG. 8.—Same tumour as in Fig. 7, 15 days after pituitary stalk section. Already the tumour is regressing. Note the similarity to Fig. 2. H. and E. ×220.

FIG. 9.—One mammary tumour of rat showing good regression 17 weeks after pituitary stalk section. H. and E. ×456.

FIG. 10.—Same rat as in Fig. 9. Another tumour showing active growth (note mitoses) 17 weeks after stalk section. H. and E. ×456.

FIG. 11.—Marked regression seen in rat's mammary tumour 8 weeks after pituitary stalk section. H. and E. ×180.

FIG. 12.—Good regression (above) and an island of non-regression (below) seen in a mammary tumour of a rat 12 weeks after pituitary stalk section. H. and E. ×178.

FIG. 13.—Mammary tumour of rat still active 4 weeks after pituitary stalk section. Biopsy specimen taken at the time of a second operation, in which the pituitary was actually removed (see Fig. 14). H. and E. ×450.

FIG. 14.—Same tumour as in Fig. 13, showing striking regression 11 days after the total hypophysectomy had been performed. H. and E. ×450.

Daniel and Prichard.

Daniel and Prichard.

9

10

11

12

Daniel and Prichard.

Daniel and Prichard.

after hypophysectomy. In one rat killed at 2 months, the new tumour, which was adjacent to the remnant of a regressed tumour, was an adenoma of characteristic histological appearance with numerous mitoses. In the other rat, which was killed at 11 weeks after operation when 2 tumours had regressed, the new tumour was not characteristic, histologically, of the adenomata of this series. The nuclei of the cells were small and crowded together. No mitoses were seen and the tissue did not look active.

In one of the twenty rats subjected to hypophysectomy, the animal's single tumour continued to enlarge after operation ; it ulcerated and consequently the rat was killed 16 days after hypophysectomy. Histologically the tumour was found to show a very unusual picture, the cells being small and closely packed. A very few mitoses were seen. The appearance was completely different from the typical active adenomatous tumour seen in the biopsy specimen, but it did not resemble a characteristic regressing tumour.

In three animals, with tumours of the typical adenomatous type which regressed after hypophysectomy, a fibro-adenoma also developed elsewhere. In one of these rats the fibro-adenoma had appeared before operation and continued to enlarge afterwards ; in the other two rats the fibro-adenomata appeared a few weeks after operation.

*Effect of pituitary stalk section*

Thirty-seven rats with well established mammary tumours were subjected to the operation of pituitary stalk section. Eighteen of these animals made a satisfactory recovery from the operation and survived long enough to show the effect which it had had on the tumours. To some extent the effect could be assessed by macroscopic observation and measurement of the tumours. Some tumours shrank rapidly (as rapidly as after hypophysectomy) ; other tumours continued to grow, while others again showed no obvious change in size. However, as in the case of hypophysectomised rats, our assessment as to whether regression occurred or not was based on a histological study of the tumours (Table I).

In none of the rats was there complete regression of its tumour or tumours. However, some regression occurred in 11 animals. The tumours of these rats showed a mixed picture similar to that seen in some of the hypophysectomised rats, with only individual tumours or parts of tumours regressing (Fig. 9 and 12), while other tumours in the same animal, or even parts of the regressing tumours, showed either continued activity or a static condition (Fig. 10 and 12). In 4 of the 11 rats the greater part of the tumour or tumours showed regression, but in the remaining 7 rats the areas of regression were estimated to comprise less than one half of the tumour tissue. In at least 7 of these 11 rats the regression seen in an individual tumour was quite as pronounced as any regression found after hypophysectomy (see Fig. 7, 8 and 11), and histologically the features of the regressing tumours were similar.

The tumours of the remaining 7 of the 18 rats submitted to stalk section showed no indication of regression after the operation.

*Pituitary stalk section followed by hypophysectomy*

Two rats, whose tumours had continued to grow after pituitary stalk section (Fig. 13) were subjected to hypophysectomy 4 and 10 weeks respectively after the first operation. A few earlier attempts had shown that rats did not tolerate this second operation well, and accordingly these two animals were given 5 mg. prednisolone trimethylacetate (Ultracortenol ; Ciba) intramuscularly for 3 days, starting the day before hypophysectomy. After the hypophysectomy the tumours in both rats decreased in size, and histologically at 11 days and 8 weeks respectively, showed the characteristic picture of regression (Fig. 14).

### DISCUSSION

To understand the logical basis for using the operation of pituitary stalk section as a means of inhibiting the growth of hormone-dependent tumours, it is necessary to know the main anatomical features of the pituitary gland and the effect which transection of the pituitary stalk has upon it, particularly on pars distalis (anterior lobe). It is now generally recognised that the secretion of many hormones by the cells of pars distalis is brought about by a neuro-humoral mechanism controlled from the hypothalamus. The cells of certain of the hypothalamic nuclei send nerve fibres down the pituitary stalk and into the infundibular process (neural or posterior lobe). Many of these fibres end on vessels which form a primary capillary bed in the median eminence, the stalk and the infundibular process, and there liberate their " neuro-secretion ". As we have shown elsewhere (Xuereb, Prichard and Daniel, 1954*a*, *b* ; Daniel and Prichard, 1956, 1957*a*) it is from this primary capillary bed that the hypophysial portal vessels, which alone supply pars distalis, take their origin. These portal vessels, therefore, carry to the cells of pars distalis the neuro-humors which they require for the secretion of hormones. When the pituitary stalk is cut, and a barrier is placed between the cut ends, the long portal vessels which run down the stalk to supply the greater part of pars distalis are severed ; consequently a large infarct develops in the centre of this lobe (Daniel and Prichard, 1957*b*, 1958 ; Daniel *et al.*, 1958 ; Adams *et al.*, 1963*a*, *b*, *c*, *d*, *e*). However, the circulation through the short portal vessels, which are situated below the level of the transection, remains unimpaired, and thus the territory which they supply along the dorsal and caudal borders of pars distalis survive. But since the hypothalamic-hypophysial nerve tract, passing down the pituitary stalk to the infundibular process, is also severed when the stalk is cut, these short portal vessels are denervated, and consequently, although they keep alive certain parts of pars distalis, they no longer carry any humoral substances coming directly from the hypothalamus. A narrow rim of cells along the ventral and lateral borders of pars distalis also survives, presumably preserved by the adjacent highly vascular dura, and this rim equally receives no direct influence from the hypothalamus.

Thus the effect of transection of the pituitary stalk on pars distalis is two-fold : over a wide area the parenchymal cells actually die, while in the remaining parts of the lobe the cells, although preserved, are deprived of the direct hypothalamic influences which they need for the secretion of hormones. There are therefore good theoretical grounds for believing that this operation must very seriously impair the function of pars distalis, and indeed experiments on normal animals have shown this to be the case. After transection of the stalk, growth is retarded, and there is a marked atrophy of the adrenal glands and of the gonads, to mention only a few of the effects which we have observed (Adams *et al.*, 1963*a*). Thus at the outset of the present study it seemed probable that pituitary stalk section would inhibit the growth of hormone-dependent tumours, but the question remained as to whether the control of tumour growth resulting from this operation would be as complete as that achieved by actual removal of the pituitary gland. Within their limited sphere the experiments reported here provide an answer to this question. For in these particular rats, with hormone-dependent mammary tumours induced by 3-methylcholanthrene, the operation of pituitary stalk section proved to be less effective than complete hypophysectomy in causing regression of the tumours.

Our findings in regard to the response of the tumours to hypophysectomy confirm those of Huggins *et al.* (1959), who reported that out of 9 rats with tumours induced by 3-methylcholanthrene all 9 showed a decrease in the size of the tumours after hypophysectomy. Similarly, Dao and Sunderland (1959) obtained a decrease in size of the tumours in 6 out of 6 rats after the same operation. Numerically, our results, as set out in Table I, would suggest that in a rather larger series of experiments than those of these previous workers we obtained a less high percentage of tumour regression. However, it should be stressed that our assessment of regression was based on a histological examination of the tumours. If our results had been assessed in terms of a decrease in the size of the tumours, the proportion of our rats showing tumour regression after hypophysectomy would have been 95 per cent. For in 19 out of 20 rats of the hypophysectomised series all the tumours present at operation, excluding the occasional fibro-adenoma, decreased in size. But in 6 of these 19 animals a histological study of the remnant of the tumours showed an occasional small island where the tumour tissue had not regressed, and for this reason we have excluded these animals from the group showing complete regression. Moreover, in two of these six rats a new tumour developed some weeks post-operatively. In the last of the 20 rats the single tumour continued to increase in size after hypophysectomy ; histologically, at 16 days after operation its appearance was much altered from the typical active tumour seen in the biopsy specimen, but it did not resemble the picture of regressing tumours seen in other rats at a comparable stage after operation, and we therefore feel that in this animal it should be deemed that no regression had occurred.

The operation of pituitary stalk section did produce some very good instances of regression, but fewer of the tumours regressed. Both these facts were evident macroscopically as well as histologically. Although there was some regression of the tumours in 11 of the 18 rats in this group, and in some instances the regression was dramatic (see Fig. 7 and 8), there was no animal in which its tumour or tumours regressed throughout. In the other 7 rats the tumours showed no sign of regression.

A further indication that removal of the pituitary is more effective than pituitary stalk section in causing the tumours to regress is probably to be seen in the two experiments in which hypophysectomy was carried out on rats in which pituitary stalk section had failed to produce regression of the tumours (Fig. 13). After this second operation the tumours regressed (Fig. 14). However, it is possible that the prednisolone which was given to these rats as replacement therapy for the immediate post-operative period may have contributed to the regression.

From the results of the hypophysectomy experiments it is clear, as Huggins and his colleagues (1959) found, that most of the tumours induced by 3-methylcholanthrene are hormone-dependent. However, an occasional tumour, or part of a tumour, did not regress after removal of the pituitary, suggesting that another agency controlled these particular tumour cells. In comparing the effectiveness of pituitary stalk section with that of hypophysectomy, it must therefore be borne in mind that some tumour tissue which was not hormone-dependent may well have been present in the animals subjected to stalk section. But even allowing for this, the results of the two groups of experiments do appear to indicate that the operation of pituitary stalk section was not as effective as hypophysectomy in causing regression of the tumours. It would seem that after transection of the pituitary stalk, in spite of the extensive infarction of pars distalis, the surviving portion of this lobe, although denervated and separated from the hypothalamus by an impermeable barrier, continued to have sufficient influence to maintain some tumour growth.

There is no doubt that transection of the pituitary stalk has caused regression of mammary tumours in a certain number of women who have been subjected to the operation (Ehni and Eckles, 1959). We suspect that the operation may tend to have better results in the human subject for at least one reason, namely, that the infarct produced in man involves a larger proportion of pars distalis than it does in the rat. Studies on several species of animal have indicated that the percentage of the lobe which becomes infarcted after stalk section tends to be greater in those species which have the larger pituitary glands. Thus, while in the rat the infarct occupies from 29 to 78 per cent of pars distalis (Adams *et al.*, 1963*a*, *b*), in the goat with its very much larger pituitary, the infarct involves from 69 to 90 per cent of the lobe (Adams *et al.*, 1963*c*). In the sheep, which has a gland as large as the human pituitary, from 85 to 96 per cent of pars distalis becomes infarcted (Adams *et al.*, 1963*d*), and in a human gland examined 30 hours after stalk section the infarcted area represented 90 per cent of the lobe (Adams *et al.*, 1963*e*). In these larger species, therefore, the proportion of pars distalis which survives is much smaller than it is in the rat (22–71 per cent), being only 10–31 per cent in the goat, 4–15 per cent in the sheep, and 10 per cent in man. Clearly the smaller the remnant of tissue which survives, the less is likely to be the output of hormones, and the above figures indicate that the residual hormonal activity present in the larger species, including man, must be considerably less than it is in the rat.

### SUMMARY

Mammary tumours, predominantly hormone-dependent in type, were induced in rats by feeding with 3-methylcholanthrene. One group of rats with tumours was subjected to hypophysectomy; in another group the pituitary stalk was transected and a barrier inserted between the cut ends. The effect of the operations on the tumours was assessed histologically. Hypophysectomy proved to be more effective than pituitary stalk section in causing regression of the tumours, although some striking examples of regression were seen after the latter operation.

The rationale, based on anatomical and experimental data, for using the operation of pituitary stalk section as an alternative to hypophysectomy for the control of tumours is briefly discussed. The findings in the present study suggest that in the rat subjected to stalk section, despite the extensive infarction of pars distalis, the amount of parenchyma which survives is large enough to maintain some tumour growth. It is suggested that in man pituitary stalk section might be more nearly equivalent to hypophysectomy in inhibiting tumour growth, since the proportion of pars distalis which escapes infarction when the stalk is cut is considerably smaller than it is in the rat.

We wish to express our thanks to Mr. E. Bernard and Miss J. Booty for much help with the animals, and to Mrs. J. Storms and Miss C. Haseler for their technical assistance. The work was supported by a grant from the British Empire Cancer Campaign, for which we are very grateful.

### REFERENCES

ADAMS, J. H., DANIEL, P. M. AND PRICHARD, M. M. L.—(1963*a*) *Quart. J. exp. Physiol.*, **48**, 217.—(1963*b*) *J. Physiol.*, **165**, 22 *P*.—(1963*c*) *J. Path. Bact.* (in press).—(1963*d*) *Acta endocr., Copenhagen*, Suppl. 81 (in press).

*Iidem* AND SCHURR, P. H.—(1963*e*) *J. Physiol.* **166**, 39 *P*.

DANIEL, P. M. and PRICHARD, M. M. L.—(1956) *Quart. J. exp. Physiol.*, **41**, 215.—(1957*a*) *Ibid.*, **42**, 237.—(1957*b*) *Ibid.*, **47**, 248.—(1958) *Amer. J. Path.*, **34**, 433.—(1961) *Brit. J. Cancer*, **15**, 828.

*Iidem* AND SCHURR, P. H.—(1958) *Lancet*, i, 1101.

DAO, T. L. AND SUNDERLAND, H.—(1959) *J. nat. Cancer Inst.*, **23**, 567.

EHNI, G. AND ECKLES, N. E.—(1959) *J. Neurosurg.*, **16**, 628.

HUGGINS, C., BRIZIARELLI, G. AND SUTTON, H.—(1959) *J. exp. Med.*, **109**, 25.

XUEREB, G. P., PRICHARD, M. M. L. AND DANIEL, P. M.—(1954*a*) *Quart. J. exp. Physiol.*, **39**, 199.—(1954*b*) *Ibid.*, **39**, 219.